# Machine Learning-Based Ensemble Feature Selection and Nested Cross-Validation for miRNA Biomarker Discovery in Usher Syndrome

**DOI:** 10.3390/bioengineering12050497

**Published:** 2025-05-08

**Authors:** Rama Krishna Thelagathoti, Dinesh S. Chandel, Wesley A. Tom, Chao Jiang, Gary Krzyzanowski, Appolinaire Olou, M. Rohan Fernando

**Affiliations:** Molecular Diagnostic Research Laboratory, Center for Sensory Neuroscience, Boys Town National Research Hospital, Omaha, NE 68010, USA; ramakrishna.thelagathoti@boystown.org (R.K.T.); wesley.tom@boystown.org (W.A.T.); chao.jiang@boystown.org (C.J.); gary.krzyzanowski@boystown.org (G.K.); appolinaire.olou@boystown.org (A.O.)

**Keywords:** ensemble feature selection, biomarker discovery, usher syndrome, miRNA, machine learning, nested cross-validation

## Abstract

Usher syndrome (USH) is a rare genetic disorder affecting vision, hearing, and balance. Identifying reliable biomarkers is crucial for early diagnosis and understanding disease mechanisms. MicroRNAs (miRNAs), key regulators of gene expression, hold promise as biomarkers for USH. This study aimed to identify a minimal subset of miRNAs that could serve as biomarkers to effectively differentiate USH from controls. We employed ensemble feature selection techniques to select the top miRNAs appearing in at least three algorithms. Machine learning models were trained and tested using this subset, followed by validation on an independent 10% sample. Our approach identified 10 key miRNAs as potential biomarkers for USH. To further validate their biological relevance, we conducted pathway analysis, which revealed significant pathways associated with USH. Furthermore, our approach achieved high classification performance, with an accuracy of 97.7%, sensitivity of 98%, specificity of 92.5%, F1 score of 95.8%, and an AUC of 97.5%. These findings demonstrate that combining ensemble feature selection with machine learning provides a robust strategy for miRNA biomarker discovery, advancing USH diagnosis and molecular understanding.

## 1. Introduction

Usher syndrome is a rare genetic disorder characterized by a combination of hearing loss, progressive vision loss due to retinitis pigmentosa and, in some cases, balance issues [1,2]. It is the most common cause of inherited deaf-blindness, accounting for approximately 50% of cases where individuals experience both hearing and vision impairment [3]. Usher syndrome presents in various clinical subtypes, each with varying severity and onset of symptoms, making its diagnosis particularly complex [4]. The heterogeneity in symptoms and the various genetic mutations associated with the syndrome make diagnosis challenging, often requiring a combination of clinical assessments, audiological tests, and genetic screening [5,6].

In recent years, microRNAs (miRNAs) have emerged as promising biomarkers for diagnosing various genetic and complex diseases, including Usher syndrome [7,8]. miRNAs are small, non-coding RNA molecules that regulate gene expression and play crucial roles in various cellular processes [9,10]. Abnormal miRNA expression has been linked to a wide range of diseases, including cancers, neurological disorders, and genetic syndromes [10]. Given their stability in biological fluids and their specificity to certain pathological states, miRNAs have gained attention as potential biomarkers for early and accurate diagnosis. In the context of Usher syndrome, profiling miRNA expression can offer insights into the molecular mechanisms underlying the disorder and potentially aid in its detection [7,11].

While miRNAs hold significant promise for diagnosis, not all miRNAs contribute equally to disease progression. Identifying a minimal subset of miRNAs that are most relevant to disease is critical for both diagnostic accuracy and biological understanding [12,13,14]. A smaller miRNA set improves the interpretability of diagnostic models, aiding in better understanding the disease’s molecular underpinnings [15,16,17]. Clinically, a minimal miRNA set reduces the complexity and cost of diagnostic tests, making them more feasible for large-scale or routine screening [18]. Moreover, focusing on a smaller, highly relevant miRNA subset facilitates the development of targeted therapies and personalized treatments [15].

Feature selection techniques are instrumental in reducing the dimensionality of miRNA datasets by identifying the most relevant miRNAs associated with any disease, including Usher syndrome [12,13,15]. Given the high dimensionality of miRNA expression profiles, selecting a minimal feature set is essential. Rare genetic disorder studies often have relatively small sample sizes. Feature selection methods, such as filter, wrapper, and embedded techniques, can help identify the most impactful miRNAs [19]. Therefore, choosing a compact set of features is crucial for developing robust and interpretable models. In the case of Usher syndrome, which is also a rare genetic disorder, applying feature selection to miRNA data can help isolate the key miRNAs associated with the disorder, facilitating a more efficient and accurate diagnostic process.

Given the complexity of Usher syndrome and the vast miRNA datasets generated from expression profiling, automating the diagnostic process with machine learning classifiers is essential. In this study, we propose a machine learning-based approach that integrates ensemble feature selection and nested cross-validation to identify the minimal miRNA feature set needed for the automated detection of Usher syndrome. This study has two main objectives: (1) to design and develop an ensemble feature selection method combined with nested cross-validation to identify the minimal miRNA set for classification; and (2) to utilize a range of supervised machine learning classifiers to train, test, and validate the models to identify the best-performing one. This approach not only reduces the time and labor involved in manual diagnosis, but also improves the accuracy and reliability of predictions. Furthermore, nested cross-validation is particularly beneficial when working with small datasets, where data acquisition is often a challenge.

## 2. Relevant Work

Ensemble feature selection has proven to be a promising approach in miRNA-based disease classification, particularly for high-dimensional datasets. Studies highlight its ability to identify minimal miRNA subsets that improve diagnostic accuracy and reduce dimensionality. For example, Cai et al. (2015) employed an ensemble method with multiple classifiers to differentiate lung cancer samples from controls, achieving enhanced robustness in classification [20]. Similarly, Sarkar et al. (2021) leveraged machine learning integrated with survival analysis to identify breast cancer subtype-specific miRNA biomarkers, demonstrating improved diagnostic and prognostic precision [15]. These methods underscore the value of ensemble strategies in addressing the challenges of high-dimensional biological data.

Lopez-Rincon et al. (2020) [21] applied an ensemble recursive feature selection approach to identify circulating miRNAs as biomarkers for cancer classification across various tumor types. This method improved the interpretability and reliability of cancer classification by pinpointing a minimal subset of miRNAs relevant to different tumor types [21]. In a related study, Lopez-Rincon et al. (2019) [12] developed an ensemble feature selection framework to identify a 100-miRNA signature for cancer classification. Their automated feature selection approach demonstrated the scalability of ensemble feature selection in cancer classification. However, the large number of selected features (100 miRNAs) may not be suitable for diseases associated with fewer miRNAs, such as rare genetic disorders [12]. Colombelli et al. (2022) [22] proposed a hybrid ensemble feature selection method for identifying miRNA biomarkers from transcriptomic data. This hybrid design enabled a more comprehensive selection of candidate biomarkers, enhancing model accuracy and interpretability [22].

The current study overcomes the limitations of previous research by incorporating an adaptive ensemble feature selection method combined with nested cross-validation for miRNA-based classification, specifically targeting Usher syndrome. This novel approach ensures robust feature selection and validation, addressing overfitting by employing nested cross-validation throughout both the feature selection and classification processes. Furthermore, our method dynamically updates the minimal feature set as new data becomes available, making it particularly well-suited for rare genetic disorders like Usher syndrome, which often suffer from small sample sizes. This integration of dynamic adaptability and scalability represents a significant advancement in miRNA-based disease classification, offering a more reliable and flexible framework than prior methods.

## 3. Materials and Methods

**Figure 1:**
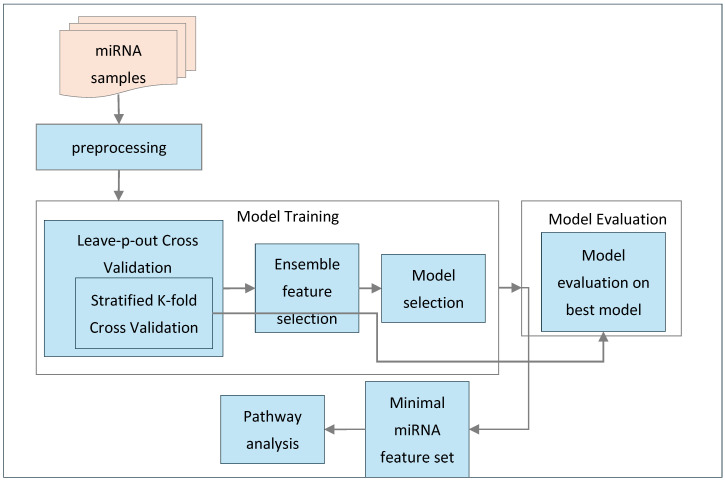
Overview of the methodology.

This section outlines the methodology employed in this study. As shown in Figure 1, the process begins with preprocessing the miRNA samples, followed by feature selection and model training using an ensemble feature selection approach combined with nested cross-validation. This methodology helps identify the best-performing model and the minimal subset of miRNA features. The selected model is then evaluated and validated using these features to assess its performance. Additionally, the minimal set of miRNAs is used for pathway analysis to extract relevant biological pathways. Furthermore, this section provides an overview of the feature selection techniques and machine learning classification algorithms used in this study.

### 3.1. miRNA Samples Extraction and Quantification

For each sample in the study, total miRNA was extracted using methods described previously [7]. Briefly, QIAzol reagent was used to isolate total RNA, and miRNA was samples in this study were collected from patient derived B-lymphocyte cell lines. MiRNA was purified from cell lines using miRNeasy Tissue/Cells Advanced Micro Kit (QIAGEN Sciences Inc., Germantown, MD, USA). MicroRNA expression was quantified for all samples using NanoString nCounter Human v3 miRNA Expression assays (cat. # CSO-MIR3-12, Bruker Corp., Billerica, MA, USA). Quality control and batch normalization of miRNA count data was performed using NAnostring quality Control dasHbOard (NACHO) package in R [23]. This resulted in a miRNA count matrix for 798 miRNAs for 60 samples, which was utilized for subsequent machine learning analysis. There were 31 Usher samples, and 29 control samples int the cohort.

### 3.2. Feature Selection

Feature selection, also known as feature reduction or variable subset selection, is the process of identifying a minimal subset of the most relevant features from a larger set of features in a dataset [12,24]. When working with high-dimensional data, such as miRNA data, it becomes essential to select a small, optimal subset that captures the critical information of the entire dataset without significant loss of detail. This process is fundamental in building robust learning models. The importance of feature selection has been widely studied in fields like bioinformatics and pattern recognition [25,26]. In general, feature selection techniques are designed to minimize overfitting and enhance model performance, leading to improved predictive accuracy in supervised classification and better cluster detection in clustering tasks. Furthermore, they contribute to the creation of faster and more cost-efficient models while also providing valuable insights into the underlying processes that produced the data [16,27].

In the realm of classification, feature selection methods can be classified into three categories based on their selection strategy of the features: filter methods, wrapper methods, and embedded methods. Filter methods select the relevance of features based on certain properties such as statistical significance. Features are selected before choosing any machine learning model. These techniques are computationally efficient and help eliminate irrelevant features, allowing for a simplified model without risking overfitting [28]. Wrapper methods involve using a specific machine learning algorithm to evaluate the performance of different feature subsets by training the model repeatedly. This approach often yields better feature selection tailored to the model, but it can be computationally expensive [29]. Embedded methods integrate feature selection within the model training process, allowing for simultaneous feature selection and model optimization. This approach typically results in models that are both efficient and accurate, as they consider feature interactions during the selection process [30].

When working with miRNA expression profile datasets, selecting the most relevant features (miRNAs) is essential for constructing accurate and interpretable models [12]. Due to the high dimensionality of miRNA datasets and the typically small sample sizes, effective feature selection methods can greatly enhance model performance and mitigate the risk of overfitting [24]. Research has shown that ensemble feature selection methods combined with cross-validation techniques can effectively identify an optimal minimal subset of features. This approach enhances model performance and ensures robust validation on unseen data while reducing the risk of overfitting, outperforming single-feature selection techniques [15]. In this study, we propose an ensemble feature selection approach combined with nested cross-validation to identify the minimal miRNA feature set for classification.

### 3.3. Ensemble Feature Selection Algorithms

Ensemble feature selection identifies an optimal feature set by combining results from multiple individual feature selection algorithms [31]. This approach differs from single-feature selection strategies, which identify key features using a singular method. For example, single methods might filter out low-variance features or recursively eliminate those that do not contribute to model performance. In contrast, ensemble selection integrates optimal feature sets from various techniques to find the best overall feature set. Previous studies shows that ensemble learning results in more robust classification outcomes and produces a superior optimal feature set compared to single-feature selection approaches [12,15,16,17,20,21,22]. In this study, we selected four distinct feature selection methods, each representing a different category of techniques, to ensure a comprehensive approach to feature selection. Recursive Feature Elimination (RFE), taken from wrapper methods, Random Forest feature importance (RF), an embedded method, the k-best method (k-best), a filter technique, and Least Absolute Shrinkage and Selection Operator (LASSO) to introduce regularization by penalizing certain coefficients.

#### 3.3.1. Recursive Feature Elimination

Recursive Feature Elimination (RFE) is a wrapper-based feature selection method that iteratively selects the most important features by recursively removing the least important ones [32]. The principle of RFE involves training a model using all available features, evaluating their importance scores, and then recursively eliminating the least important features until the desired number of features is reached [33]. The goal of RFE is to identify an optimal subset of features from the complete list of features in the dataset. Initially, a supervised learning model is trained using the full feature set to predict the target values. Afterward, the importance of each feature is evaluated based on the model’s learned parameters, with less important features identified by their scores. The feature with the lowest importance score is then eliminated from the dataset. This process continues iteratively, with the model being retrained after each removal, until the desired number of features is selected.

#### 3.3.2. Random Forest Feature Importance

Random Forest (RF) feature importance belong to embedded feature selection family that evaluates the significance of each feature based on its contribution to the prediction in a Random Forest model [34]. The primary goal of RF feature importance is to identify and rank features based on their predictive power, allowing for the selection of the most influential features. A Random Forest model is trained by constructing multiple decision trees using bootstrap samples from the dataset. During the training process, the importance of each feature is evaluated based on its contribution to the model’s predictive accuracy, typically using metrics such as Mean Decrease Gini (MDG) or Mean Decrease Accuracy (MDA) [35,36]. This method effectively selects important features while retaining the benefits of the Random Forest’s robustness against overfitting.

#### 3.3.3. Least Absolute Shrinkage and Selection Operator (LASSO)

LASSO is a regression analysis technique that combines both variable selection and regularization to improve the prediction accuracy and interpretability of a statistical model. It works by minimizing a specific objective function, which includes a regularization term. In LASSO regression, L1 regularization is applied, which adds a penalty to the model based on the absolute values of the coefficients. This regularization encourages sparsity in the model by pushing the coefficients of less important features towards zero. As the regularization parameter increases, more coefficients are driven towards zero, effectively removing less relevant predictors from the model. The result is a simpler and more interpretable model that tends to generalize better when applied to unseen data. This makes LASSO a powerful tool for feature selection and model simplification [37].

#### 3.3.4. K-Best Feature Selection

K-Best is a filter-based feature selection method that assesses the relevance of each feature with respect to the target variable. The process starts by evaluating all features in the dataset using statistical tests like the Chi-squared test, ANOVA F-value, or mutual information. The goal is to identify the top k features that provide the most useful information for predicting the target variable. The selection process can be outlined as follows:

1. For each feature, compute a score using a chosen statistical measure.
2. Sort the features based on their scores in descending order.
3. Select the top k features with the highest scores.

K-Best feature selection helps reduce the dimensionality of the dataset by removing less informative features. This results in improved model performance while retaining the most relevant features for predictive modeling [28].

### 3.4. Overview of Machine Learning Techniques

This section presents the list of ML techniques we have utilized in this study including Logistic Regression (LR), Random Forest (RF), Support Vector Machine (SVM), Extreme Gradient Boosting (XGB), and Ada Boost (ADB).

#### 3.4.1. Logistic Regression

Logistic regression is a statistical method used for binary classification, where it models the relationship between a dependent binary variable and one or more independent variables. The method estimates probabilities using the logistic function [38]. In this model, the probability that the target variable is equal to 1, given the features, is calculated. The coefficients for each feature are estimated by maximizing the likelihood function, which measures how well the model fits the observed outcomes [39]. Logistic Regression can be used to classify miRNA expression data by estimating the probability that a given miRNA profile corresponds to a specific disease class. Its simplicity and ability to handle binary outcomes make it a valuable tool for predicting the presence of disease based on miRNA expression levels.

#### 3.4.2. Random Forest

Random Forest is an ensemble learning method used for classification. It builds multiple decision trees from bootstrapped samples of the dataset and averages their predictions [40]. For a given dataset, a Random Forest creates multiple decision trees, each using a random subset of features. During training, the algorithm minimizes the Gini impurity or entropy at each node to effectively split the data [35,36]. Random Forest is particularly effective for miRNA data classification due to its ability to handle high-dimensional datasets and its robustness against overfitting. It identifies important miRNAs by averaging predictions from multiple decision trees, making it a useful tool for classifying diseases based on miRNA profiles.

#### 3.4.3. Support Vector Machine (SVM)

A Support Vector Machine (SVM) is a supervised learning algorithm that constructs a hyperplane in a high-dimensional space to separate different classes [41]. It aims to find the optimal hyperplane that maximizes the margin between the classes. The SVM is particularly effective for classification problems with complex and high-dimensional data, such as miRNA expression profiles. The SVM is ideal for distinguishing between disease and healthy profiles due to its ability to handle non-linear relationships. The kernel trick allows SVMs to capture these non-linearities by transforming the input space into a higher-dimensional feature space, making it a powerful method for miRNA data classification [42].

#### 3.4.4. Extreme Gradient Boosting (XGBoost)

XGBoost is an optimized gradient boosting algorithm that builds models in a sequential manner, where each new model corrects the errors made by the previous ones. It aims to improve the performance of weak learners by iteratively adjusting the model to enhance prediction accuracy [43]. The algorithm’s objective function combines the loss function and a regularization term, ensuring that the model is both accurate and simple, avoiding overfitting. XGBoost is highly effective for miRNA classification tasks, as it boosts the performance of weak learners by iteratively improving prediction accuracy. Additionally, its ability to handle missing values and irregular data makes it a strong choice for miRNA-based disease classification, allowing it to effectively work with complex datasets [44].

#### 3.4.5. Adaptive Boosting (AdaBoost)

AdaBoost is an ensemble learning method that combines multiple weak classifiers to form a strong classifier [45]. It works by training classifiers sequentially, with each new classifier focusing on the errors made by the previous ones. The algorithm assigns weights to each instance, increasing the weights of misclassified instances at each iteration, helping the model improve over time. The final model is a weighted sum of the predictions from each weak classifier, where each classifier’s weight is determined by its accuracy. AdaBoost is particularly useful in miRNA classification because it enhances the model’s ability to predict disease. It improves the accuracy of classifiers, even when miRNA data are complex or imbalanced, making it a strong choice for disease classification tasks where data may be noisy or challenging [46].

### 3.5. Ensemble Feature Selection with Nested Cross-Validation

In this section, we introduce the ensemble feature selection approach combined with nested cross-validation, a robust methodology designed to identify the most relevant features while minimizing overfitting. Cross-validation is a technique that involves partitioning data into multiple subsets. Some of these subsets are utilized for training the model, while the others are reserved for testing or validation. This process continues until every subset has served as both a training and validation set [41]. A commonly used method is k-fold cross-validation, where the dataset is divided into k-equally sized subsets (folds). In this method, the model is trained on k-1 folds and tested on the remaining fold. This procedure is repeated k times, ensuring that each fold is tested at least once [47]. By employing different data subsets for training and testing, cross-validation offers a more accurate estimate of a model’s performance on unseen data [48]. Nested cross-validation involves multiple levels of cross-validation, often structured as an outer loop and an inner loop [49]. This technique helps reduce overfitting and enhances overall model performance.

Following algorithm describes our proposed ensemble feature selection with nested cross-validation as shown in Algorithm 1.

The methodology begins with the input of a miRNA expression dataset, which contains both the samples and the associated features necessary for classification. The first step involves initializing key variables: *N*, the number of samples; *F*, the number of miRNA features; and an empty set called *F_minimal_* to store the minimal set of miRNA features identified during the analysis. Additionally, a variable M* is initialized to hold the best-performing model after evaluation. Two sets are initialized to facilitate the nested cross-validation process: outer training sets, which consists of the outer training sets (T1, T2, …, Tp), and validation sets, which includes the corresponding validation sets (V1, V2, …, Vp).

**Figure 2:**
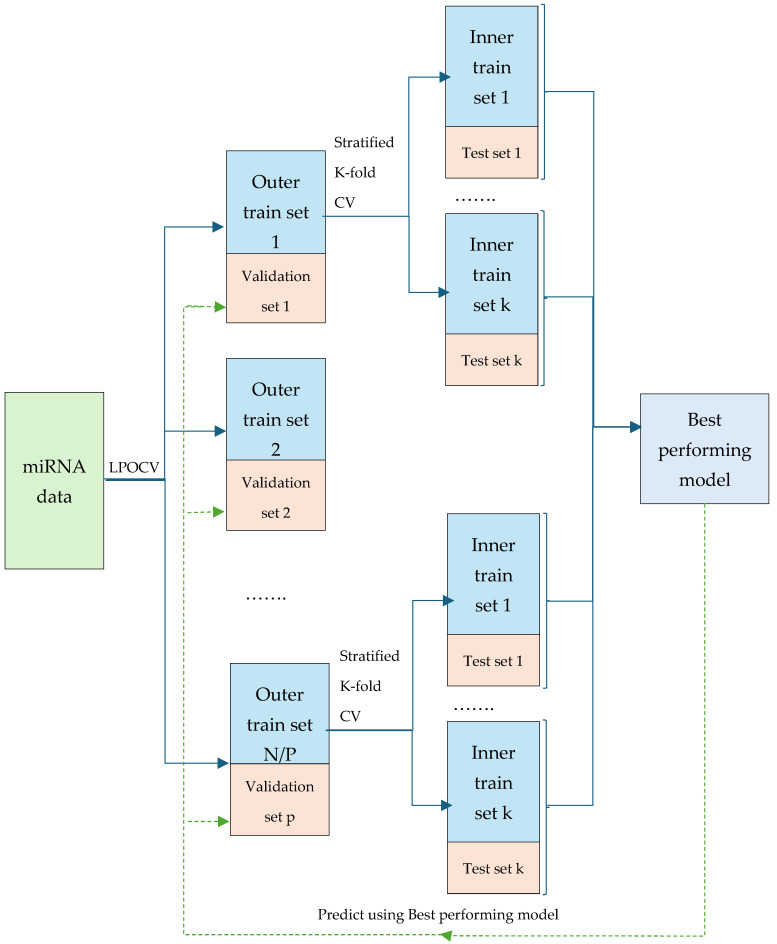
Overview of ensemble feature selection with nested cross-validation.

The second step involves executing Leave-P-Out Cross-Validation (LPOCV) as shown in Figure 2, where the input data are divided into outer training and validation sets. This method generates multiple splits, with each outer training set (Ti) containing 90% of the total data, while the corresponding validation sets (Vi) consist of the remaining 10%. This process ensures that each instance in the dataset is eventually used for validation, providing a robust evaluation framework for model performance.
**Algorithm 1:** Ensemble feature selection with nested cross-validation**Input**: miRNA expression dataset ***D*** ∈ ℝ^*N* × *F*^, where *N* is the number of samples and *F* is the number of miRNA features.
**Output:**
      •Minimal miRNA feature set *F*_minimal_      •Best-performing model M*      •Mean performance metrics across all validation sets
**Step 1. Initialization**
             *F*_minimal_ ← ∅
             M* ← None
             $\mathrm{Let}\;p=\frac{N}{10}$ (i.e., Leave-6-Out Cross-Validation when *N* = 60)
             Generate *p* = 10 non-overlapping folds:
                  ${\left\{(T_{i},V_{i})\right\}}_{i=1}^{p}$, where *T*_*i*_ ∈ ℝ^(*N* − *p*) × *F*^, *V_i_* ∈ ℝ^*p* × *F*^
**Step 2. Outer Cross-Validation (Leave-p-Out)**
             **for** each *i* ∈ {1, 2, …, *p*} **do**
                  Let *T*_*i*_ be the outer training set and *V*_*i*_ be the outer validation set
**Step 3. Inner Cross-Validation (Stratified k-Fold) and Feature Selection**
                  $\mathrm{Split}\;T_{i}\;\mathrm{into}\;k\;\mathrm{stratified}\;\mathrm{folds}:\;{\left\{(t_{j},v_{j})\right\}}_{j=1}^{k}$
                  **for** each *j* ∈ {1, 2, …, *k*} **do**
                       **Feature Selection on**
*t_j_*: 
                            Apply RFE, Random Forest importance, LASSO, and SelectKBest
                       **Model Training:**

                            Train classifiers (LR, RF, SVM, XGBoost, AdaBoost) on *t_j_*
                       **Model Evaluation:**
                            Evaluate on *v_j_* using Accuracy, Sensitivity, Specificity, F1 Score, and AUC
                       **Model Selection:**

                            Choose best-performing model *M_j_*
                       **Update Feature Set:**

                            Add features to *F*_minimal_ if selected in ≥ 3 inner folds
                  Select most frequent model across inner folds as:
                       M* = argmax_*Mj*_ (frequency of selection in inner folds)
**Step 4. Model Validation on Outer Fold**
                      Use M* and *F*_minimal_ to classify *V_i_*
                      Evaluate performance using Accuracy, Sensitivity, Specificity, F1 Score, and AUC
**Return:**-*F*_minimal_—final feature set-M*—best-performing model-Average performance metrics over all *V_i_*, *i* = 1, 2, …, *p*

In the third step, the focus shifts to the inner cross-validation and feature selection process. For each outer training set Ti, stratified k-fold cross-validation is employed to further divide the data. As shown in Figure 2, each outer training set (Ti) is split into multiple inner training sets (t1, t2, …, ti) and corresponding inner test sets (v1, v2, …, vi). Feature selection is then performed on each inner training set using various methods, such as Recursive Feature Elimination (RFE), Random Forest (RF) feature importance, LASSO, and the k-best method using the ANOVA F-score as the statistical indicator to rank features based on their discriminative power. Simultaneously, different classifiers, including Logistic Regression (LR), Random Forest (RF), Support Vector Machines (SVM), Extreme Gradient Boosting (XGB), and AdaBoost (ADB), are trained on these inner training sets. The performance of each model is evaluated against its corresponding inner test set using key metrics like accuracy, sensitivity, specificity, F1 score, and AUC. The model that achieves the best mean performance across all metrics for the current outer training set is designated as M*. In addition to that, miRNA features that consistently appear at least 3 times across all iterations are appended to the *F_minimal_*.

The final step involves validating the selected model and feature set. For each validation set Vi, the M* identified from the inner cross-validation is used to perform classification, utilizing the minimal miRNA feature set derived from the previous step. The performance of this model is then evaluated on the validation sets using the same metrics: accuracy, sensitivity, specificity, F1 score, and AUC.

Ultimately, the output of this comprehensive methodology includes the minimal miRNA feature set consistently selected across all inner k-folds, the best-performing model based on mean performance across all inner train and test sets, and the mean metrics of this best model evaluated against the validation sets. This structured approach ensures a thorough and reliable framework for identifying significant miRNA features and achieving accurate classification outcomes.

### 3.6. Finding Enriched Pathways

#### 3.6.1. miRNA Gene Target Prediction

TargetScanHuman version 8.0 was used to predict genes with binding sites matching the 10 miRNAs from the model, resulting in 6115 genes with matching miRNA target sites [50]. Of these, 572 gene targets had cumulative weighted context scores (CWCS) less than −0.5, indicating strong gene suppression. All 6115 unique gene targets were included in gene ontology enrichment analysis and metabolic pathway analysis [50].

#### 3.6.2. Gene Ontology Enrichment and Pathway Analysis

The R package ‘clusterProfiler’ was used to identify gene ontologies (GOs) and pathways which might be influenced by miRNAs from the model [51]. For gene ontology enrichment analysis, the ‘enrichGO()’ function was used with an adjusted p-value cutoff of 0.05 and a minimum gene set size of 5. R’s ‘enrichR’ package was used to identify pathways affected by miRNAs using the ‘enrichr()’ function against the Reactome pathway database [52,53]. Putative affected pathways were determined to be significant at an adjusted *p*-value threshold of 0.05.

## 4. Results

This section presents the results in four key aspects that highlight the effectiveness of our proposed approach. First, we present the minimal miRNA biomarker set identified from the ensemble feature selection method. Second, we highlight the best model selected during the training phase and its performance metrics. Third, evaluation of selected best model and its performance on validation sets. Finally, we present the biological pathway analysis based on the selected miRNA features.

### 4.1. Selected Minimal miRNA Feature Set

We identified a minimal set consisting of 10 miRNAs through the ensemble feature selection method combined with nested cross-validation. These miRNAs significantly contribute to the classification of Usher syndrome when compared to control samples. The selected miRNAs are: hsa-miR-148a-3p, hsa-miR-183-5p, hsa-miR-146a-5p, hsa-miR-28-5p, hsa-miR-30c-5p, hsa-miR-551b-3p, hsa-miR-642a-5p, hsa-miR-181a-5p, hsa-miR-28-3p, hsa-miR-182-5p.

**Figure 3:**
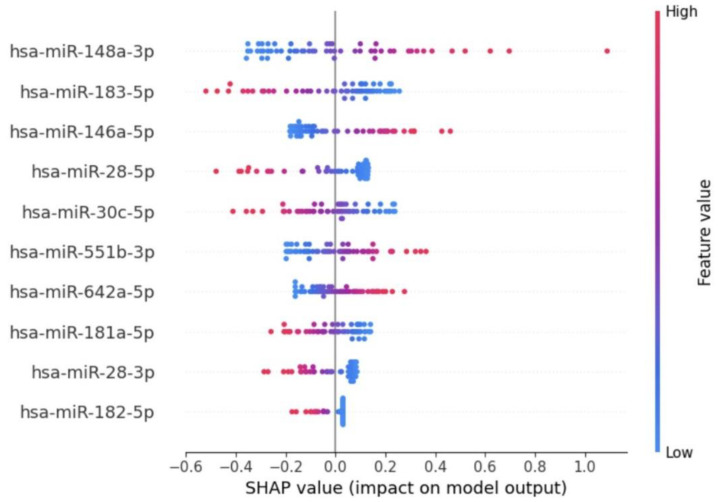
SHAP summary plot with selected miRNA features.

**Figure 4:**
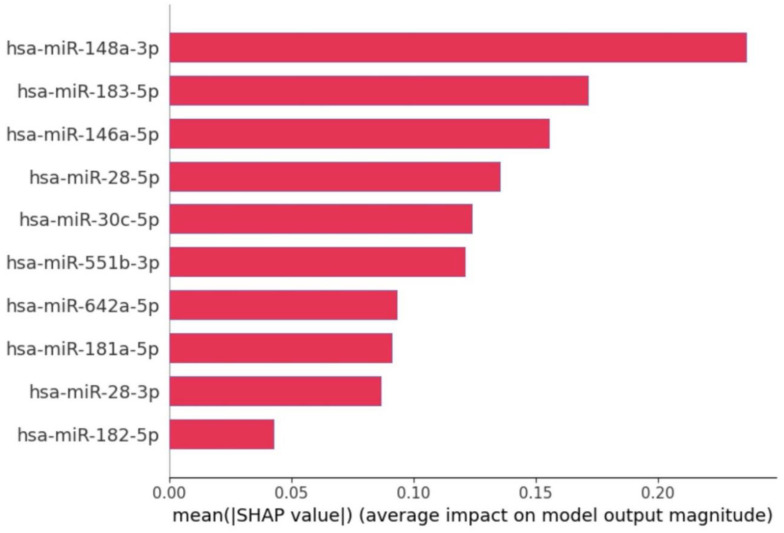
SHAP feature importance plot that shows importance of each selected feature.

The SHAP summary plot shown in Figure 3, illustrates the contribution of specific miRNAs to the model’s output for distinguishing between Usher syndrome and control samples. Each dot represents a SHAP value for a given miRNA in a sample, with color indicating the miRNA’s expression level (blue for low and red for high). The position on the *x*-axis shows the impact of the feature on the model’s prediction: positive values push the model towards predicting Usher syndrome, while negative values push towards control. The feature importance plot (in Figure 4) reveals the contribution of individual miRNAs to the classification decision between Usher syndrome patients and control samples.

Key observations for each miRNA:

hsa-miR-148a-3p: Highly ranked in SHAP feature importance, contributing significantly toward USH prediction with high SHAP values. In other words, higher expression levels (red) increase the SHAP value positively, strongly contributing to Usher syndrome prediction.
hsa-miR-183-5p: Both high and low expression values are seen across the SHAP value spectrum, indicating variable contributions. However, overall miRNA expression levels are downregulated in usher compared to control.
hsa-miR-146a-5p: Contributes to usher prediction with positive SHAP values. Expression levels are generally upregulated in usher and downregulated in control.
hsa-miR-28-5p, hsa-miR-28-3p, and hsa-miR-182-5p: These miRNAs exhibit similar expression pattern where positive SHAP pushes towards usher prediction and negative SHAP values indicates significant contribution towards control prediction. Moreover, Lower SHAP values indicate that expression levels are significantly downregulated in usher compared to control.
hsa-miR-551b-3p and hsa-miR-642a-5p: Show a consistent pattern of positive SHAP values, indicating their importance in usher prediction. These miRNAs are upregulated in usher and downregulated in control.
hsa-miR-30c-5p and hsa-miR-181a-5p: Both miRNAs were expressed at higher levels in controls and reduced in usher, contributing negatively to SHAP values for control classification.

### 4.2. Model Training Results

For model training, five machine learning classifiers were employed for classifying Usher syndrome using miRNA data: Logistic Regression (LR), Random Forest (RF), Support Vector Machine (SVM), XGBoost (XGB), and AdaBoost (ADB). We used the default hyperparameters for all machine learning models as provided by their respective libraries, including the default number of trees for Random Forest and the default kernel function for SVM. No systematic hyperparameter tuning (e.g., grid search or random search) was performed in the current implementation. To ensure robust model evaluation, LPOCV approach with *p* = 6 (10% of total samples) was implemented, resulting in 10 iterations. At each iteration, 54 samples were used for training, and 6 samples were held out for validation. For the inner training, the 54 training samples were split into 80% for training and 20% for testing using stratified k-fold cross-validation. Important features were selected during model training and testing, with final model performance evaluated using the validation sets. Table 1 illustrates the performance metrics of the five classifiers on training and testing sets, including accuracy, sensitivity, specificity, F1 score, and AUC, while Table 2 displays the evaluation metrics of the best-performing model on the validation sets. They are computed using standard classification metrics [13] derived from the confusion matrix, which are described below.


**Predicted Positive**

**Predicted Negative**

**Actual Positive**
TPFN
**Actual Negative**
FPTN

Where:

- TP = True Positives (real positives predicted as positives);
- FN = False Negatives (real positives incorrectly predicted as negatives);
- FP = False Positives (real negatives incorrectly predicted as positives);
- TN = True Negatives (real negatives correctly predicted as negatives).

Performance Metric Formulas

1. Accuracy

$$Accuracy=\frac{\left(TP+TN\right)}{\left(TP+TN+FP+FN\right)}$$

2. Sensitivity (Recall or True positive rate)

$$Sensitivity=\frac{TP}{\left(TP+FP\right)}$$

3. Specificity

$$Specificity=\frac{TN}{\left(TN+FP\right)}$$

4. F1 score

$$F1\;score=\frac{2*\;(Precision*Recall)}{\left(Precision+Recall\right)}$$

where $Precision=\frac{TP}{\left(TP+FP\right)}$.

5. Area Under the Curve (AUC)

Calculated from the ROC curve plotting True Positive Rate (Sensitivity) against False Positive Rate (FPR), where

$$FPR=\frac{FP}{\left(FP+TN\right)}$$

Accuracy

As shown in Table 1, Logistic Regression achieved the highest mean accuracy (0.98 ± 0.05, CI: 0.95–1.00), and consistently performed well across all iterations, maintaining values between 0.95 and 1.00. SVM also achieved a mean accuracy of 0.98 ± 0.05 (CI: 0.95–1.00), while Random Forest followed closely with 0.97 ± 0.07 (CI: 0.92–1.00). XGBoost (0.93 ± 0.15) and AdaBoost (0.95 ± 0.15) showed greater variability, with AdaBoost displaying the most instability, sometimes dropping to accuracies as low as 0.84. Despite this, AdaBoost occasionally achieved accuracy as high as 1.0.

Sensitivity

As shown in Table 1, Logistic Regression and Random Forest maintained perfect sensitivity (1.00 ± 0.00) across all iterations, clearly outperforming the other models. SVM showed strong but slightly lower sensitivity (0.95 ± 0.00). XGBoost and AdaBoost were the least stable, with sensitivity ranging from 0.67 to 1.0, and a large standard deviation of ±0.30, indicating inconsistency across iterations.

Specificity

Regarding specificity, presented in Table 1, AdaBoost achieved perfect specificity (1.00 ± 0.00), followed by XGBoost (0.97 ± 0.10), Logistic Regression and SVM (0.97 ± 0.10), and Random Forest (0.93 ± 0.13). However, AdaBoost displayed the highest variance, with performance fluctuating significantly. Logistic Regression, in contrast, maintained a consistent specificity with lower variance, reinforcing its reliability.

F1 Score

As shown in Table 1, Logistic Regression exhibited the most consistent performance with F1 scores of 0.99 ± 0.04 (CI: 0.95–1.00), reflecting robust model behavior. SVM and Random Forest also performed well (both at 0.97–0.99) with minor fluctuations. XGBoost had a lower mean F1 score (0.89 ± 0.30), and AdaBoost showed the most variability (0.90 ± 0.30), occasionally dipping below 0.90. Among the classifiers, Logistic Regression was the most stable, making it a potentially more reliable choice for miRNA-based classification tasks.

AUC

As shown in Table 1, AUC values for Logistic Regression remained nearly perfect (0.99 ± 0.10, CI: 0.99–1.00) across iterations, indicating high and consistent discriminatory power. SVM and Random Forest also showed strong AUC values (0.99 ± 0.03). XGBoost and AdaBoost exhibited more variability, though both reached a maximum AUC of 1.00 in certain iterations.

Overall, Logistic Regression demonstrated the most consistent and robust performance across accuracy, sensitivity, specificity, F1 score, and AUC. In contrast, AdaBoost exhibited the most unstable results, often lagging behind other models in terms of accuracy, sensitivity, and specificity. These results suggest that Logistic Regression may be the most suitable model for miRNA-based Usher syndrome classification, offering reliable performance with minimal fluctuations across different iterations of the LPOCV process.

**Table 1:** Model training results including mean ± std with 95% confidence interval).

Model	Accuracy	Sensitivity	Specificity	F1 Score	AUC
Logistic Regression	0.98 ± 0.05 (0.95, 1.00)	1.00 ± 0.00 (1.00, 1.00)	0.97 ± 0.10 (0.89, 1.00)	0.99 ± 0.04 (0.95, 1.00)	0.99 ± 0.10 (0.99, 1.00)
Random Forest	0.97 ± 0.07 (0.92, 1.00)	1.00 ± 0.00 (1.00, 1.00)	0.93 ± 0.13 (0.83, 1.00)	0.97 ± 0.06 (0.93, 1.00)	0.95 ± 0.03 (0.90, 1.00)
SVM	0.98 ± 0.05 (0.94, 1.00)	0.95 ± 0.00 (0.90, 1.00)	0.97 ± 0.10 (0.89, 1.00)	0.99 ± 0.04 (0.95, 1.00)	0.99 ± 0.03 (0.93, 1.00)
XGBoost	0.93 ± 0.15 (0.82, 1.00)	0.90 ± 0.30 (0.67, 1.00)	0.97 ± 0.10 (0.96, 1.00)	0.89 ± 0.30 (0.66, 1.00)	0.99 ± 0.03 (0.96, 1.00)
AdaBoost	0.95 ± 0.15 (0.84, 1.00)	0.90 ± 0.30 (0.67, 1.00)	0.91 ± 0.00 (0.80, 1.00)	0.90 ± 0.30 (0.91, 0.96)	0.94 ± 0.13 (0.90, 1.00))

**Table 2:** Model validation results including mean ± std with 95% confidence interval).

Model	Accuracy	Sensitivity	Specificity	F1 Score	AUC
Logistic Regression	0.97 ± 0.08 (0.93, 1.00)	0.98 ± 0.07 (0.91, 1.00)	0.93 ± 0.16 (0.91, 1.00)	0.95 ± 0.10 (0.91, 1.00)	0.97 ± 0.08 (0.92, 1.00)
Random Forest	0.60 ± 0.30 (0.38, 0.81)	0.30 ± 0.42 (0.00, 0.60)	1.00 ± 0.00 (1.00, 1.00)	0.93 ± 0.12 (0.84, 1.00)	0.60 ± 0.30 (0.38, 0.81)
SVM	0.90 ± 0.21 (0.82, 1.00)	0.93 ± 0.12 (0.86, 1.00)	0.97 ± 0.07 (0.91, 1.00)	0.98 ± 0.06 (0.93, 1.00)	0.90 ± 0.21 (0.86, 1.00)
XGBoost	0.50 ± 0.19 (0.36, 0.63)	0.12 ± 0.31 (0.10, 0.35)	0.90 ± 0.31 (0.67, 1.00)	0.71 ± 0.28 (0.50, 0.91)	0.50 ± 0.19 (0.36, 0.63)
AdaBoost	0.48 ± 0.14 (0.37, 0.58)	0.34 ± 0.20 (0.00, 0.36)	1.00 ± 0.00 (1.00, 1.00)	0.52 ± 0.07 (0.46, 0.58)	0.48 ± 0.14 (0.37, 0.58)

### 4.3. Model Evaluation

The performance of five models in classifying Usher syndrome samples using miRNA data was evaluated across 10 validation sets over 10 iterations, as shown in Table 2. Logistic regression model demonstrated consistent accuracy with minimal fluctuations, highlighting its ability to generalize effectively in classifying Usher syndrome samples compared to control samples. Specificity remained close to 1.0 across most iterations, indicating the model’s reliability in identifying true positives. Sensitivity, although exhibiting slight variability, was stable enough to confirm the model’s effectiveness in correctly classifying control samples. The F1 Score, a balance between precision and recall, consistently hovered around 1.00 with minimal variation, reflecting the robust behavior of the model in maintaining a strong balance between true positive rate and precision. Additionally, the Area Under the Curve (AUC) remained nearly perfect, consistently achieving values between 0.97 and 1.0. The overall performance metrics are as follows: mean Accuracy of 97%, mean Sensitivity of 98.0%, mean Specificity of 93%, mean F1 Score of 95%, and mean AUC of 97%. Furthermore, Logistic Regression demonstrated stable and reliable performance across all evaluation metrics, making it a suitable and robust choice for miRNA-based classification of Usher syndrome.

### 4.4. Pathway Analysis

**Figure 5:**
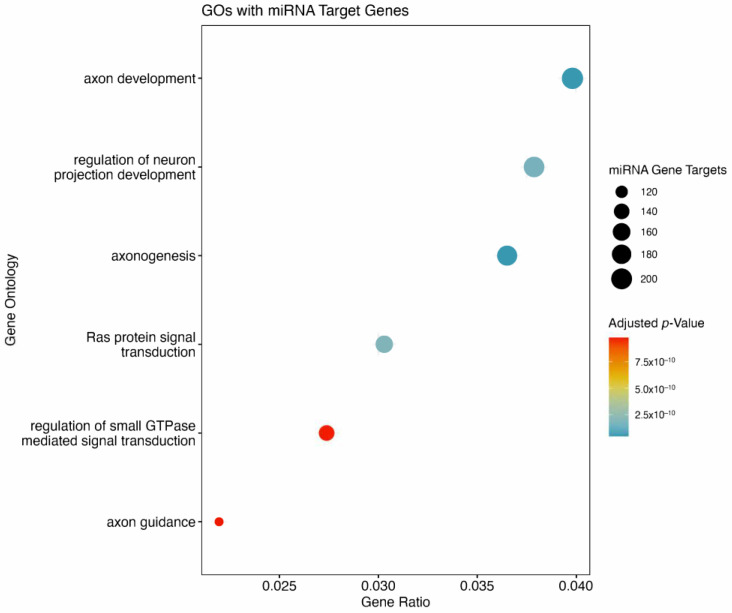
Panel A depicts gene ontologies (GOs) which contain genes targeted by miRNAs from the model. GOs were considered significant at a BH adjusted *p*-value ≤ 0.05. The size of the dot in the dot plot corresponds with the number of genes in the GO targeted by miRNAs from the model, while the color corresponds to adjusted *p*-values.

**Figure 6:**
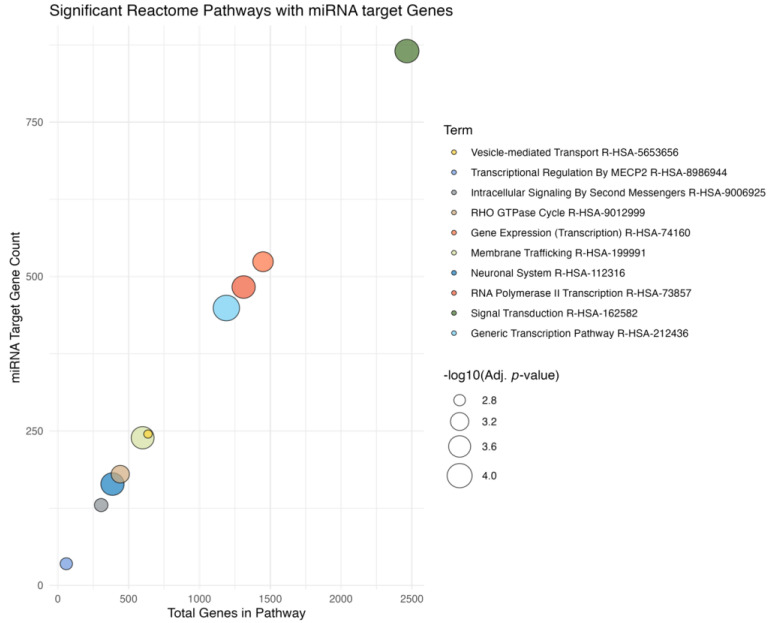
EnrichR analysis of pathways with gene targets corresponding to the miRNAs from the model, using Reactome pathways database. Y-axis is the number of miRNA associated genes, and the x-axis is the total number of genes in the pathway. Dots in the plot represent the different Reactome pathways, which may be influenced by miRNAs from the model. Pathways are colored according to Reactome ID, and the size of the dot reflects the −log10 (*p*-value).

There were 6115 unique genes predicted to be targeted by the 10 miRNAs derived from the ML model. In total, 572 of these genes were predicted to be strongly suppressed by the miRNAs of interest. There were six GOs flagged to be affected (Figure 5). Of the six GOs, four were associated with neuronal or axon development. The remaining two GOs were signal transduction related, with RAS protein signal transduction, and GTPase mediated signal transduction both predicted to be suppressed by miRNAs of interest. Included are the top 10 pathways associated with the genes targeted by miRNAs from the ML model (Figure 6). The top two pathways in terms of combined scores are both related to neuronal development, corroborating observations from GO enrichment findings (Table 3).

## 5. Discussion

The proposed methodology for biomarker discovery in Usher syndrome integrates ensemble feature selection with nested cross-validation to identify a minimal set of miRNA biomarkers. This minimal biomarker set represents the smallest subset of miRNAs that can reliably distinguish Usher syndrome from control samples. However, due to the rarity of Usher syndrome, obtaining large sample sizes remains a challenge, which may impact the generalizability of the selected biomarkers. As more data become available, this approach is designed to refine and update the biomarker set, improving its robustness and biological relevance over time. Future validation on larger datasets will enhance its clinical applicability and reliability.

By employing nested cross-validation, this methodology ensures that dataset partitioning into training and validation sets is performed iteratively. The training sets undergo additional stratified cross-validation, where multiple validation sets are tested to generate stable performance metrics. Compared to single-set validation, this process provides more reliable feature selection and performance evaluation. The combination of ensemble feature selection and rigorous cross-validation enhances the stability of identified biomarkers, ensuring their reproducibility, even with limited data.

Future studies incorporating larger sample sizes will be essential to further validate and refine the identified miRNA biomarkers, advancing their potential use in early diagnosis and therapeutic targeting of Usher syndrome. The methodology leverages LPOCV for the outer loop and stratified k-fold cross-validation for the inner loop, ensuring robust biomarker selection.

## 6. Limitations and Future Directions

While our study demonstrates promising results for the automated detection of Usher syndrome using miRNA expression profiles, there are several limitations. First, the dataset used is small, reflecting the challenges inherent to studying rare genetic disorders like Usher syndrome. A small sample size may limit the robustness and generalizability of the identified minimal feature set, which may not fully capture the variability across a more diverse population. Additionally, the heterogeneity of miRNA expression influenced by various factors (e.g., age, genetic background, and environmental factors) could impact the model’s performance when applied to different cohorts. Another limitation lies in miRNA data itself; variations in miRNA extraction and quantification techniques may influence the reproducibility of results across different labs or clinical settings.

Future research should focus on expanding the dataset by including samples from diverse populations and incorporating longitudinal data where possible. This would allow the feature selection method to capture a broader range of genetic variability, enhancing the generalizability of the model. In addition, further studies should explore integrating multi-omics data (e.g., mRNA, protein, and epigenetic data) to improve predictive accuracy and capture complex biological interactions related to Usher syndrome. Another potential avenue is the incorporation of transfer learning or domain adaptation techniques to enable the model trained on miRNA data to be effectively applied to new data sources or patient groups. Validation in a clinical setting is also necessary to assess the practicality and reliability of the proposed framework in real-world diagnostic workflows. Finally, as more samples become available, it would be valuable to explore deep learning-based models that could potentially improve classification performance by capturing non-linear relationships in the data.

## 7. Conclusions

This study presents a machine learning-based approach incorporating ensemble feature selection and nested cross-validation for the discovery of miRNA biomarkers associated with Usher syndrome. Given the rarity of Usher syndrome, obtaining large datasets is challenging. Our method aims to maximize the reliability of biomarker identification by selecting a minimal set of miRNAs that remain robust against variations in sample size. The ensemble feature selection component integrates results from multiple models, while nested cross-validation ensures rigorous evaluation. This provides more reliable biomarker selection compared to conventional validation methods. The identified minimal biomarker set represents a promising step toward developing miRNA-based biomarkers for Usher syndrome, although its generalizability to larger, more diverse datasets remains a future goal. Our results demonstrate that as more data become available, the biomarker set can be refined and updated, enhancing its clinical applicability. Overall, this study provides a robust framework for feature selection and validation in small, complex datasets, with potential applications beyond Usher syndrome to other rare genetic disorders where data availability is limited.

## Figures and Tables

**Table 3:** Top 10 pathways predicted to be influenced by miRNAs of interest. Gene-count is the number of genes in the pathway targeted by miRNAs from ML model. Genes in pathway are the total number of genes in the term pathway followed by the percent of affected genes in the pathway. P-values and adjusted p-values are provided from enrichR results, as well as the odds ratios and combined score outputs from enrichR. Significance was determined at an adjusted *p*-value < 0.05, and a combined score > 15.

Term	Gene-Count	Genes in Pathway	Percent of Path	*p*-Value	Adj. *p*-Value	Odds-Ratio	Combined-Score
Transcriptional Regulation By MECP2 R-HSA-8986944	35	60	58.33	7.56 × 10^−6^	0.0015	3.19	37.64
Neuronal System R-HSA-112316	164	386	42.49	3.91 × 10^−7^	0.0002	1.70	25.03
Generic Transcription Pathway R-HSA-212436	449	1190	37.73	3.28 × 10^−8^	0.0001	1.41	24.23
Membrane Trafficking R-HSA-199991	239	599	39.90	5.34 × 10^−7^	0.0002	1.53	22.07
RHO GTPase Cycle R-HSA-9012999	180	441	40.82	2.55 × 10^−6^	0.0007	1.58	20.39
Intracellular Signaling By Second Messengers R-HSA-9006925	130	306	42.48	6.03 × 10^−6^	0.0013	1.69	20.34
RNA Polymerase II Transcription R-HSA-73857	483	1312	36.81	3.31 × 10^−7^	0.0002	1.35	20.16
Signal Transduction R-HSA-162582	865	2465	35.09	1.47 × 10^−7^	0.0001	1.27	19.91
Gene Expression (Transcription) R-HSA-74160	524	1449	36.16	1.27 × 10^−6^	0.0004	1.31	17.83
Vesicle-mediated Transport R-HSA-5653656	245	637	38.46	9.82 × 10^−6^	0.0018	1.44	16.57

## Data Availability

The datasets generated and analyzed during the current study are available from the corresponding author upon reasonable request. Additionally, the code used for analysis is also available upon request.
